# Cardiovascular abnormalities and its correlation with genotypes of children with osteogenesis imperfecta

**DOI:** 10.3389/fendo.2022.1004946

**Published:** 2022-10-20

**Authors:** Dichen Zhao, Yongtai Liu, Jidong Liu, Jing Hu, Qian Zhang, Ou Wang, Yan Jiang, Weibo Xia, Xiaoping Xing, Mei Li

**Affiliations:** ^1^ Department of Endocrinology, National Health Commission Key Laboratory of Endocrinology, Peking Union Medical College Hospital, Chinese Academy of Medical Sciences and Peking Union Medical College, Beijing, China; ^2^ Department of Cardiology, Peking Union Medical College Hospital, Chinese Academy of Medical Sciences and Peking Union Medical College, Beijing, China; ^3^ Department of Endocrinology, Qilu Hospital, Cheeloo College of Medicine, Shandong University, Jinan, China

**Keywords:** osteogenesis imperfecta, cardiovascular abnormalities, gene mutation, type I collagen, echocardiography

## Abstract

**Background and objectives:**

Osteogenesis imperfecta (OI) is a rare disorder of abnormal production or modification of type I collagen, which is caused by mutations in *COL1A1*, *COL1A2* or other genes. We investigate the cardiac abnormalities and its correlation with pathogenic mutations in OI children.

**Methods:**

A cross-sectional comparative study was completed in a relatively large sample of OI children, who were matched in body surface area (BSA) with healthy controls. All echocardiography was performed by experienced cardiologists using Vivid 7 equipment (GE Medical Systems, Horton, Norway). The resting standard 12-lead electrocardiogram (ECG) were obtained in OI patients by FX-8600 machine. Skeletal phenotypes of OI patients were evaluated, including information of bone fractures, deformities, motility, and bone mineral density (BMD). Pathogenic mutations of OI were detected by a next-generation sequencing panel and confirmed by Sanger sequencing.

**Results:**

A total of 69 OI children and 42 healthy children matched in BSA were enrolled. Abnormalities of echocardiography were found in 6 OI children, including enlarged left atrium (n=5), increased internal diameter of the left ventricle (n=1), who all carried the *COL1A1* mutation. Mild regurgitation of mitral or tricuspid valves was observed in 26 OI patients. Abnormal ECG manifestations were found in 8 OI children, including deep Q wave, T wave change, premature ventricular complexes, short P-R interval, incomplete bundle branch block and high voltage of left ventricular. Compared with healthy controls, OI children had significant larger values in the main pulmonary artery (1.84 vs 1.60 cm, *P* < 0.01), left atrial diameter (2.58 vs 2.11 cm, *P* < 0.001), left ventricular internal dimension at end-diastolic (LVEDd) (3.85 vs 3.50 cm, *P* < 0.05) and lower left ventricular ejection fraction (LVEF) (68.40% vs 71.74%, *P* < 0.01). Moreover, OI patients with *COL1A1* mutation tended to have greater main pulmonary artery, larger diameters of left atrial and LVEDd, and lower LVEF than healthy controls. *COL1A1* mutation was correlated to dilated MPA (β = 1.557, *P* < 0.01), LAD (β = 3.915, *P* < 0.001), and LVEDd (β = 2.714, *P* < 0.01), and decreased LVEF (β = -3.249, *P* < 0.01).

**Conclusions:**

Cardiovascular alterations were identified in OI children, including increased dimensions of the main pulmonary artery and left chamber, and low LVEF. The cardiovascular abnormalities seemed to be correlated to *COL1A1* mutation and defects of type I collagen, which expanded our understandings of the cardiac phenotypes of OI children.

## Introduction

Osteogenesis imperfecta (OI) is a heterogeneous hereditary connective tissue disorder caused by genetic defects in synthesis or modification of type I collagen, which is characterized by increased bone fragility, recurrent bone fractures and progressive bone deformity (1). OI is estimated to affect about 1/13,500-15,000 neonates (2). According to clinical severity, OI is clinically categorized into five subgroups, with type I being mild, type II lethal perinatally, type III the most severe among the survivors, type IV of intermediate severity, and type V with calcification of the interosseous membranes and/or hypertrophic callus (3). In recent years, the classification of OI has evolved with advances in molecular diagnosis which results in at least 20 forms of OI according to different gene mutations (4). Other collagen-rich tissues can also be affected in OI patients, including sclera, skin, heart and tendon, and so on (5). Extra-skeletal manifestations of OI include blue sclera, dentinogenesis imperfecta, joint hypermobility, hearing impairment, cardiovascular and pulmonary complications (6).

Recently, studies have indicated that morbidity and mortality of OI are closely associated with the abnormalities in cardiovascular and pulmonary systems (7, 8). Abnormalities in the cardiovascular system are found to be common in OI patients, including valvular disease, atrial fibrillation and heart failure, and the more severe the OI phenotype, the higher the risk of cardiovascular abnormalities (9, 10). As valves, tendinous cords, fibrous rings, and the cardiac septum of hearts are mainly composed of type I and III collagen, type I collagen contributes to the stiffer and rigid property of the myocardium (11). Therefore, a decrease of the collagen was associated with a more compliant ventricle, which results in increased LV diastolic internal diameters and volume (12). Moreover, the degree of collagen crosslinking was associated with cardiac function. A more compliant and less stiff collagen fibers would hinder the force development of the myocytes and disrupted the systolic function of hearts (13). Taken together, collagen fibers in the cardiac extra cellular matrix (ECM) contribute to keeping the rigidity and the architecture of hearts (12). However, few studies investigate the cardiovascular abnormalities of OI children, and it is unclear whether the cardiovascular abnormalities have a correlation with the genotypes of OI children.

The present study aimed to investigate the cardiovascular characteristics by echocardiography and electrocardiogram in a relatively large sample of OI children. Furthermore, we tried to analyze the relationship between cardiovascular alterations with skeletal phenotypes and pathogenic gene mutations in OI children.

## Subjects and methods

This was a single-center, cross-sectional study conducted from January 2017 to January 2022 in Endocrinology Department of Peking Union Medical College Hospital (PUMCH). The patients were diagnosed as OI if they met the following criteria: (1) Patients had a history of more than one fracture under minor trauma, and with an age- and sex-adjusted BMD Z-score less than or equal to -1.0 at lumbar spine (LS) or femoral neck (FN). (2) Patients with BMD Z-scores less than or equal to -2.0 at LS or FN. (3) The diagnosis was confirmed by pathogenic mutation detection (14). Aside from abnormal hemodynamics, body size was an important affecting factors for evaluating cardiovascular structures (15). Therefore, healthy children with matched body surface areas (BSA) were included as the control group, who came to the clinic for physical examinations and completed the examinations of echocardiography. These controls had no history of cardiac diseases, skeletal diseases, chronic hepatic or kidney disorders, or other hereditary diseases. Informed consents were obtained from the parents or guardians of OI children and healthy controls.

For OI children and healthy controls, echocardiographic examinations were performed with Vivid I equipment (GE Medical Systems, Horton, Norway) using a 3.4-MHz multifrequency transducer. The echocardiography protocol consisted of complete cardiac examinations, which were performed by at least two experienced cardiologists at the same time to reduce the measurement error. The echocardiogram was interpreted by experienced cardiologists to minimize variability. The investigator did not know the clinical group of OI patients or controls. Proximal ascending aorta diameter (PAO), main pulmonary artery (MPA), right ventricular end-diastolic dimension (RVEDd), left atrial anteroposterior dimension (LAD), left ventricular end-diastolic dimension (LVEDd), interventricular septal wall thickness (IVST), LV posterior wall thickness (LVPWT), LV ejection fraction (LVEF) were measured following the Guideline of the American Society of Echocardiography (16). LAD, LVEDd, IVST, and LVPWT were measured in the parasternal long-axis view using M-mode echocardiography. LV mass (LVM) was calculated by the Devereux formula (15). RVEDd and LVEF were assessed using the modified biplane Simpson’s equation in the apical four-chamber and two-chamber views. All detectable regurgitations were included and divided into mild, moderate or severe following the recommendations of the American Society of Echocardiography (15). According to previous reports (9, 17–21), we defined the echocardiographic abnormalities as follows: (1) Increased dimensions of cardiac chambers or great arteries out of the normal range for the patient’s peer (Z-score > 2.0); (2) Moderate-severe valve regurgitation; (3) Other possible echocardiographic abnormalities (15). The resting standard 12-lead electrocardiogram (ECG) was obtained in OI patients by a FX-8600 machine (Fukuda sangyo, Japan). Resting heart rates, PR intervals, QRS duration, QRS axis, and QT intervals were examined and reviewed by experienced ECG analyzers. ECG pathologies included short PR interval, abnormal Q wave, high voltage, T wave abnormalities, the incomplete bundle branch block (RBBB) and premature ventricular complexes (PVC) (22, 23).

Demographic data of gender, age, height, and weight of OI children were collected. BSA were calculated using the Haycock formula (24). The skeletal phenotypes were evaluated, including sites and number of fractures, bone deformities, and movement ability. Serum levels of calcium (Ca), and phosphate (P) were measured by automated analyzers (ADVIA1800, Siemens, Germany). The serum level of 25-hydroxyvitamin D (25OHD) was measured using an automated electrochemiluminescence system (E170; Roche Diagnostics, Switzerland). The extra-skeletal phenotypes were evaluated carefully, including sclera, dentin, joints, and ligaments. The joint hypermobility was defined with Beighton scores (25). BMD at lumbar spine 2-4 (LS) and proximal hip of OI patients were measured by dual energy X-ray absorptiometry (DXA, Lunar Prodigy Advance, GE Healthcare, USA). BMD Z-scores of LS and FN were calculated on the basis of the reference data of BMD in Chinese and Asian children (26, 27). X-ray films of anteroposterior and lateral spines were completed and vertebral compression fracture was determined by Genant semi-quantitative method.

Pathogenic gene mutations of OI patients were identified by a next-generation sequencing (NGS) panel (Illumina HiSeq2000 platform, Illumina, Inc., San Diego, CA, USA), which covered currently known pathogenic genes of OI and 700 candidate genes of inherited bone disorders. The sequencing was performed with a coverage rate of 98.95% and an average depth of 200× on target regions in each of the chromosomes (14). To verify the gene mutation detected by NGS, polymerase chain reaction (PCR) and Sanger sequencing were conducted. According to pathogenic mutation genes, OI patients were divided into groups of *COL1A1*, *COL1A2*, or other rare gene mutations. According to the effects of pathogenic mutations on type I collagen synthesis, OI patients with *COL1A1* or *COL1A2* mutations were classified as (28): haplo-insufficiency which included mutations in *COL1A1* introducing stop codons or leading to frameshifts mutations, structural change which included mutations in *COL1A1* or *COL1A2* leading to glycine substitutions in the triple-helical domain of the type I collagen, and the rest types of mutations were pooled together into the others group.

### Statistical analysis

Continuous variables were presented as averages ± standard deviation for normal distribution (such as age, height, BSA, echocardiographic parameters and so on), or as median and ranges for abnormal distribution (such as number of fractures). Categorical variables were described as percentages, including immobility, bone deformities, blue sclera, and so on. Group differences in dichotomous variables were tested for significances using the chi-square test. Comparison of continuous variables between OI patients and controls were completed by two independent-sample *t* tests. Differences between subgroups of OI (for example, Sillence classification, and genotypes of OI) and the control group were evaluated using one-way analyses of variance. Simple linear regression model was applied to investigate which variables affected echocardiographic parameters in OI patients. The respective correlation between OI, Sillence types, OI genotypes, and echocardiographic measurements, which adjusted for age, gender, and BSA were analyzed by multiple linear regression analysis.

All tests were two-tailed and a *P* value less than 0.05 was considered statistically significant. Statistical analyses were performed using SPSS software of version 26.0 (SPSS Inc., Chicago, IL, USA).

## Results

### Baseline characteristics of the subjects

A total of 69 OI patients and 42 BSA-matched healthy children were included. Baseline characteristics were shown in **Table 1**. According to the Sillence classification, there were 42 patients with OI type I, 6 with type III, 17 with type IV, and 4 with type V. The mean age of OI patients was 9.2 ± 4.3 years at receiving echocardiographic examinations, and 41 (60.8%) patients were males. The control group had 28 boys and 14 girls, with the mean age of 8.7 ± 5.1 years. BSA were similar between OI patients and the control (1.12 versus 1.09 m^2^, *P* = 0.751).

**Table 1:** Demographic data and baseline characteristics of OI patients and controls.

	OI-Total	OI-Type I	OI-Type III/IV/V	Controls
Number of patients	69	42	27	42
Gender, male/female	41/28	28/14	13/14	28/14
Age ( ± SD), year	9.2 ± 4.3	8.7 ± 4.3	10.1 ± 4.4	8.7 ± 5.1
Age at diagnosis of OI ( ± SD), year	5.7 ± 3.9	6.2 ± 4.0	5.4 ± 4.1	/
Height ( ± SD), cm	131.7 ± 24.5	134.1 ± 26.2	128.4 ± 21.9	132.1 ± 30.5
Weight ( ± SD), kg	35.0 ± 17.8	37.2 ± 20.1	32.0 ± 13.7	33.6 ± 19.7
BSA ( ± SD), m^2^	1.12 ± 0.39	1.16 ± 0.43	1.06 ± 0.31	1.09 ± 0.45
Immobility, *n* (%)	25 (36.2)^***^	5 (11.9)^***^	20 (74.1)^***,###^	0 (0)
Number of fractures, median (range)	3 (0, 100)	3 (0, 10)	4 (1, 100)	/
Bone deformities, *n* (%)	21 (30.4)^***^	1 (2.4)	20 (74.1)^***,###^	0 (0)
Scoliosis, *n* (%)	15 (21.7)	5 (11.9)	10 (37.0)^#^	/
Vertebral compression fractures, *n* (%)	33 (47.8)	15 (35.7)	18 (66.7)^#^	/
Calcium ( ± SD), mmol/L	2.49 ± 0.11	2.47 ± 0.10	2.53 ± 0.12	/
Phosphorus ( ± SD), mmol/L	1.67 ± 0.16	1.68 ± 0.15	1.67 ± 0.18	/
25-hydroxyvitamin D ( ± SD), ng/mL	31.63 ± 15.28	33.55 ± 17.33	28.54 ± 10.91	/
Lumbar spines 2-4 BMD ( ± SD), g/cm^2^	0.397 ± 0.150	0.451 ± 0.131	0.317 ± 0.148^###^	/
Lumbar spines 2-4 BMD Z-score ( ± SD)	-2.4 ± 1.8	-1.9 ± 1.6	-3.4 ± 1.8^##^	/
Femoral neck BMD ( ± SD), g/cm^2^	0.396 ± 0.157	0.465 ± 0.122	0.312 ± 0.158^###^	/
Femoral neck BMD Z-score ( ± SD)	-4.1 ± 2.6	-3.1 ± 1.7	-5.2 ± 3.1^##^	/
Blue sclera, *n* (%)	57 (82.6)	39 (92.9)	18 (66.7)^##^	/
Dentinogenesis imperfecta, *n* (%)	8 (11.6)	3 (7.1)	5 (18.5)	/
Joints hypermobility, *n* (%)	36 (52.2)	21 (50.0)	15 (55.6)	/
Cardiac diseases related symptoms/history, *n* (%)	1 (1.4)^a^	1 (2.4)	0 (0)	0 (0)

Data shown as mean or median values as appropriate. OI, osteogenesis imperfecta; BSA, body surface area; BMD, bone mineral density.

^***^P < 0.001, vs controls.

^#^P < 0.05, vs OI-type I; ^##^P < 0.01, vs OI-type I; ^###^P < 0.001, vs OI-type I.

a One patient had an atrial septal defect after birth, which self-healed at 2 years old.

Skeletal and extra-skeletal manifestations of OI children were displayed in **Table 1**. The mean age of the patients was 5.7 ± 3.9 years at diagnosis, with the median fractures number of 3. There were 25 (36.2%) patients with limited mobility, 21 (30.4%) with bone deformities, and 15 (21.7%) patients with scoliosis. Blue sclera (82.6%) and joints hypermobility (52.2%) were common extra-skeletal manifestations of OI children.

### Echocardiographic and electrocardiogram abnormalities of OI patients

An atrial septal defect was found in 1 OI patient after birth, which self-healed at 2 years old, and no obvious heart disease was found in OI patients. However, we observed 6 (8.7%) OI patients with asymptomatic cardiac abnormalities during echocardiography examinations. Among them, 1 patient had an increased internal diameter of LV, and 5 patietns with enlarged LA. Detailed information of the 6 patients were displayed in **Table 2**, 2 of them with Sillence type IV, the other 4 patients with type I. All 6 patients carried the *COL1A1* mutation, including 2 missense mutations (c. 2560G>A, p. Gly854Ser; c.1003G>A, p.Gly335Ser), 2 frameshift mutations (c.2739delT, p.Gly914Argfs*; c.1001delC, p.Pro334Leufs*), 1 nonsense mutation (c.3076C>T, p.Arg1026*), and 1 splice site mutation (c.751-2A>C). There were 26 (37.7%) OI patients with mild mitral or tricuspid valve regurgitation, which was higher than controls (4.8%, *P* < 0.001) and required no intervention. No moderate to severe valve regurgitation was found in OI children.

**Table 2:** Phenotypes and genotypes of OI patients with echocardiographic abnormalities.

No	Sex	Age (y)	BSA (m^2^)	Cardiovascular manifestations			Genotypes			Skeleton phenotypes						Extra-skeleton phenotypes		
				Cardiac- related symptoms	ECG	Echocardiography abnormalities	Mutation gene	Variation	Type I collagen change mechanism	Sillence type	Number of fractures	Mobility	Bone deformities	Vertebral compression fractures	Scoliosis	Blue sclera	Joint hypermobility	Dentinogenesis imperfecta
1	M	2.5	0.58	Asymptomatic	Nodal tachycardia, HR 128 bpm	Increased proportion of left atrium	*COL1A1*	c.2560G>A, p.Gly854Ser	Triple-helical structure change	I	2	Automatic activity	No	No	No	Yes	No	Yes
2	M	5.5	0.75	Asymptomatic	Normal, HR 92 bpm	Increased left atrium	*COL1A1*	c.2739delT, p.Gly914Argfs*	Haplo-insufficiency	I	2	Automatic activity	No	No	No	Yes	Yes	No
3	M	9	1.34	Asymptomatic	Normal, HR 97 bpm	Increased left atrium	*COL1A1*	c.3076C>T, p.Arg1026*	Haplo-insufficiency	IV	4	Wheelchair	Yes	Yes	Yes	Yes	Yes	No
4	F	10.5	1.63	Asymptomatic	Sinus arrhythmia, HR 75 bpm	Increased internal diameter of left ventricular	*COL1A1*	c.1001delC, p.Pro334Leufs*	Haplo-insufficiency	I	3	Wheelchair^#^	No	Yes	No	Yes	Yes	No
5	M	11	1.57	Asymptomatic	Sinus arrhythmia, HR 82 bpm	Increased left atrium	*COL1A1*	c.751-2A>C	Other	I	2	Automatic activity	No	Yes	No	Yes	No	No
6	M	16	1.38	Asymptomatic	Sinus arrhythmia, HR 88 bpm	Increased left atrium	*COL1A1*	c.1003G>A, p.Gly335Ser	Triple-helical structure change	IV	3	Wheelchair	Yes	Yes	Yes	Yes	Yes	No

OI, osteogenesis imperfecta; No: number; ECG, electrocardiogram; HR, heart rate; BSA, body surface area; Gly, glycine; Ser, serine; del, deletion; Arg, arginine; fs, frameshift; Pro, proline; Leu, leucine.

^#^: This patient suffered a newly-onset left fibula fracture.

Interestingly, OI patients had larger internal dimensions of the main pulmonary artery, atrium and ventricle of heart than healthy controls (**Table 3**), with significant differences in MPA (1.84 versus 1.60 cm, *P* = 0.001), LAD (2.58 versus 2.11 cm, *P* < 0.001), and LVEDd (3.85 versus 3.50 cm, *P* = 0.004). LVEF was lower in OI patients than controls (68.40% versus 71.74%, *P* = 0.003), whereas no differences were observed in PAO, RVEDd, IVST, LVPWT, or LVM. These differences remained when OI patients were grouped according to the severity of OI, including mild (Sillence type I) and moderate-severe (Sillence type III/IV/V) patients. Compared with the control group, MPA (1.86 versus 1.60 cm, *P* = 0.004), LAD (2.61 versus 2.11 cm, *P* < 0.001) and LVEDd (3.90 versus 3.50 cm, *P* = 0.024) were larger and LVEF was lower, especially in mild OI patients. No obvious differences of echocardiographic measurements were found between mild and moderate-severe OI patients.

**Table 3:** Echocardiographic parameters in OI patients and controls.

	OI-total (n=69)	OI-Type I (n=42)	OI-Type III/IV/V (n=27)	Controls (n=42)
PAO (cm)	2.10 ± 0.43	2.10 ± 0.45	2.10 ± 0.42	2.03 ± 0.45
MPA (cm)	1.84 ± 0.30^***^	1.86 ± 0.31^**^	1.81 ± 0.29^*^	1.60 ± 0.32
LAD (cm)	2.58 ± 0.51^***^	2.61 ± 0.52^***^	2.55 ± 0.51^**^	2.11 ± 0.42
LVEDd (cm)	3.85 ± 0.58^**^	3.90 ± 0.64^*^	3.78 ± 0.49	3.50 ± 0.62
RVEDd (cm)	1.77 ± 0.37	1.77 ± 0.38	1.78 ± 0.37	1.65 ± 0.22
IVST (cm)	0.56 ± 0.13	0.57 ± 0.12	0.56 ± 0.14	0.67 ± 0.27
LVPWT (cm)	0.55 ± 0.13	0.56 ± 0.13	0.54 ± 0.13	0.67 ± 0.27
LVM (g)	59.28 ± 30.93	61.68 ± 33.10	55.87 ± 27.83	66.17 ± 46.04
HR (bpm)	90.31 ± 16.00	89.86 ± 16.42	90.89 ± 15.68	91.81 ± 20.14
LVEF (%)	68.40 ± 4.49^**^	68.22 ± 4.69^**^	68.65 ± 4.26^*^	71.74 ± 5.87

Data were expressed as mean ± standard deviation. OI, osteogenesis imperfecta; PAO, proximal ascending aorta; MPA, main pulmonary artery; LAD, left atrial anteroposterior dimension; LVEDd, left ventricular end-diastolic dimension; RVEDd, right ventricular end-diastolic dimension; IVST, interventricular septal wall thickness; LVPWT, left ventricular posterior wall thickness; LVM: left ventricular mass; HR, heart rate; LVEF, left ventricular ejection fraction. Data were expressed as mean ± SD.

^*^P < 0.05, vs controls; ^**^P < 0.01, vs controls; ^***^P < 0.001, vs controls.

8 (11.6%) OI patients were detected with ECG abnormalities, including deep Q wave (n = 2), T wave change (n = 2), PVC (n = 1), short P-R interval (n = 1), incomplete RBBB (n = 1) and high voltage of LV (n = 1). 5 patients were Sillence type I, and the other 3 patients with OI type III, IV, and V, respectively. Pathogenic OI mutations of these patients were composed of *COL1A1* (n = 5), *COL1A2* (n = 1), *P3H1* (n = 1) and *IFITM5* (n = 1).

### Relationship of echocardiographic alterations and genotypes of OI patients

Pathogenic variants were detected in 57 OI patients, 39 patients with *COL1A1* mutation, 10 with *COL1A2* mutation, and 8 with other rare genes mutations (*IFITM5* n = 4, *P3H1* n = 2, *WNT1* n = 1, *FKBP10* n = 1). Compared to BSA-matched controls, OI patients with *COL1A1* mutation had greater internal diameters of MPA (1.83 versus 1.60 cm, *P* = 0.002), LAD (2.62 versus 2.11 cm, *P* < 0.001), LVEDd (3.90 versus 3.50 cm, *P* = 0.011), and lower LVEF (68.00% versus 71.74%, *P* = 0.008) (**Table 4**). After exclusion of the 6 OI patients with increased dimensions of cardiac chambers out of the normal range, parameters of heart chambers still increased in the *COL1A1* mutation group than control group. However, the echocardiographic parameters of patients with *COL1A2* mutation or other rare gene mutations showed no significant differences from control group. No differences were detected between any two of the three OI subgroups with different genotypes.

**Table 4:** Echocardiographic parameters in OI patients with different genotypes and defects of type I collagen compared with controls.

	OI patients with different genotypes			OI patients with different defects of type I collagen			Controls (n=42)
	*COL1A1* mutation (n=39)	*COL1A2* mutation (n=10)	Other rare gene mutations (n=8)	Haplo-insufficiency (n=21)	Triple-helical structure change (n=15)	Others (n=13)	
PAO (cm)	2.09 ± 0.43	2.10 ± 0.58	2.27 ± 0.30	2.14 ± 0.42	2.01 ± 0.39	2.02 ± 0.47	2.03 ± 0.45
MPA (cm)	1.83 ± 0.31^**^	1.78 ± 0.29	1.89 ± 0.21	1.84 ± 0.31^*^	1.91 ± 0.30^*^	1.75 ± 0.35	1.60 ± 0.32
LAD (cm)	2.62 ± 0.51^***^	2.55 ± 0.49	2.58 ± 0.53	2.60 ± 0.47^**^	2.73 ± 0.58^**^	2.52 ± 0.49	2.11 ± 0.42
LVEDd (cm)	3.90 ± 0.59^*^	3.78 ± 0.62	3.94 ± 0.45	3.96 ± 0.64^*^	3.90 ± 0.61	3.63 ± 0.57	3.50 ± 0.62
RVEDd (cm)	1.78 ± 0.38	1.62 ± 0.34	1.96 ± 0.29	1.71 ± 0.33	1.83 ± 0.38	1.73 ± 0.45	1.65 ± 0.22
IVST (cm)	0.57 ± 0.13	0.53 ± 0.12	0.61 ± 0.15	0.58 ± 0.13	0.56 ± 0.13	0.54 ± 0.14	0.67 ± 0.27
LVPWT (cm)	0.56 ± 0.13	0.52 ± 0.12	0.60 ± 0.12	0.58 ± 0.14	0.54 ± 0.12	0.54 ± 0.14	0.67 ± 0.27
LVM (g)	61.34 ± 32.51	51.75 ± 26.12	68.88 ± 30.96	65.21 ± 35.65	58.73 ± 26.05	51.44 ± 31.33	66.17 ± 46.04
HR (bpm)	90.47 ± 17.43	96.38 ± 15.87	87.63 ± 4.93	89.20 ± 15.97	93.77 ± 20.18	92.83 ± 17.34	91.81 ± 20.14
LVEF (%)	68.00 ± 4.40^**^	70.30 ± 4.30	68.13 ± 4.48	69.00 ± 4.64	69.21 ± 4.19	66.00 ± 3.95^**^	71.74 ± 5.87

Data were expressed as mean ± standard deviation. OI, osteogenesis imperfecta; PAO, proximal ascending aorta; MPA, main pulmonary artery; LAD, left atrial anteroposterior dimension; LVEDd, left ventricular end-diastolic dimension; RVEDd, right ventricular end-diastolic dimension; IVST, interventricular septal wall thickness; LVPWT, left ventricular posterior wall thickness; LVM, left ventricular mass; HR, heart rate; LVEF, left ventricular ejection fraction.

^*^P < 0.05, vs controls; ^**^P < 0.01, vs controls; ^***^P < 0.001, vs controls.

According to the effects of gene mutation on synthesis of type I collagen, OI patients with *COL1A1* or *COL1A2* mutation were further divided into three subgroups, including haplo-insufficiency of type I collagen (n = 21), triple-helical structure change (n = 15), and others (n = 13). Patients with haplo-insufficiency of type I collagen had larger MPA (1.84 versus 1.60 cm, *P* = 0.039), LAD (2.60 versus 2.11 cm, *P* = 0.002), and LVEDd (3.96 versus 3.50 cm, *P* = 0.037) than BSA-matched controls. Patients with structure change of type I collagen had larger diameters in MPA (1.91 versus 1.60 cm, *P* = 0.011), and LAD (2.73 versus 2.11 cm, *P* = 0.006) than control group (**Table 4**). While LVEF only showed significant difference in the group with other defects of type I collagen from control group (66.00% versus 71.74%, *P* = 0.008).

As shown in **Table 5**, age, male, and BSA had significantly positive correlation with echocardiographic parameters, except for LVEF. Diagnosis of OI showed a significant relationship with dilated MPA (β = 2.341, *P* < 0.001), LAD (β = 4.754, *P* < 0.001), LVEDd (β = 3.532, *P* = 0.004) and lower LVEF (β =-3.341, *P* = 0.001). *COL1A1* mutation had a correlation with larger MPA (β = 1.565, *P* = 0.022), LAD (β = 3.796, *P* < 0.001), LVEDd (β = 2.807, *P* = 0.031) and lower LVEF (β = -3.269, *P* = 0.003). This correlation was not found in patients with *COL1A2* or other rare genes mutations. After adjusted for age, gender, and BSA, OI, *COL1A1* mutation was still significantly correlated with larger internal dimensions of MPA (β = 1.557, *P* = 0.001), LAD (β = 3.915, *P* < 0.001), LVEDd (β = 2.714, *P* = 0.001), and lower LVEF (β = -3.249, *P* = 0.004) (**Table 6**).

**Table 5:** Factors associated with echocardiographic parameters.

	Independent variable β (95% CI)						
Dependent variable	Age	Male	BSA	OI	*COL1A1* mutation	*COL1A2* mutation	Other genes mutations
PAO	0.699 (0.576, 0.821)^***^	2.551 (0.857, 4.246)^**^	7.649 (6.194, 9.104)^***^	0.706 (-1.021, 2.433)	0.214 (-1.629, 2.057)	0.230 (-2.862, 3.322)	2.074 (-1.367, 5.515)
MPA	0.513 (0.419, 0.608)^***^	1.496 (0.201, 2.792)^*^	5.942 (4.888, 6.995)^***^	2.341 (1.122, 3.561)^***^	1.565 (0.236, 2.895)^*^	0.479 (-1.813, 2.771)	2.403 (-0.122, 4.927)
LAD	0.816 (0.660, 0.972)	2.108 (0.001, 4.214)	9.066 (7.237, 10.896)^***^	4.754 (2.865, 6.643)^***^	3.796 (1.708, 5.884)^***^	1.253 (-2.476, 4.982)	3.074 (-1.069, 7.217)
LVEDd	1.040 (0.870, 1.203)^***^	2.541 (0.079, 5.003)^*^	12.041 (10.224, 13.858)^***^	3.532 (1.167, 5.897)^**^	2.807 (0.258, 5.355)^*^	0.142 (-4.241, 4.525)	3.597 (-1.261, 8.455)
RVEDd	0.466 (0.338, 0.595)^***^	0.683 (0.874, 2.240)	5.858 (4.477, 7.238)^***^	1.236 (-0.412, 2.885)	0.841 (-0.670, 2.353)	-1.322 (-3.673, 1.029)	2.400 (-1.191, 4.991)
IVST	0.303 (0.246, 0.361)^***^	1.057 (0.255, 1.858)^*^	3.430 (2.778, 4.081)^***^	-1.053 (-1.913, -0.192)^*^	-0.585 (0.172, -1.430)	-0.748 (-2.068, 0.573)	0.164 (-1.323, 1.652)
LVPWT	0.304 (0.247, 0.362)^***^	0.994 (0.133, 1.075)^*^	3.480 (2.835, 4.125)^***^	-1.169 (-2.025, -0.312)^**^	-0.594 (-1.435, 0.248)	-0.810 (-2.124, 0.503)	0.069 (-1.414, 1.551)
LVM	6.356 (5.377, 7.335)^***^	16.117 (0.329, 31.904)^*^	73.895 (63.244, 84.545)^***^	-6.888 (-24.094, 10.318)	-1.986 (-18.447, 14.475)	-12.003 (-37.480, 13.473)	7.186 (-21.405, 35.777)
LVEF	0.194 (-0.026, 0.415)	-0.407 (-2.559, 1.745)	1.604 (-0.923, 4.132)	-3.341 (-5.350, -1.332)^**^	-3.269 (-5.430, -1.107)^**^	0.973 (-2.818, 4.764)	-1.583 (-5.826, 2.661)

BSA, body surface area; OI, osteogenesis imperfecta; PAO, proximal ascending aorta; MPA, main pulmonary artery; LAD, left atrial anteroposterior dimension; LVEDd, left ventricular end-diastolic dimension; RVEDd, right ventricular end-diastolic dimension; IVST, interventricular septal wall thickness; LVPWT, left ventricular posterior wall thickness; LVM, left ventricular mass; LVEF, left ventricular ejection fraction.

β, Regression coefficient. ^*^P < 0.05; ^**^P < 0.01; ^***^P < 0.001.

**Table 6:** Correlation between OI genotypes and echocardiographic parameters.

	Dependent variable β								
Independent variable	PAO	MPA	LAD	LVEDd	RVEDd	IVST	LVPWT	LVM	LVEF
OI	0.409	2.279^***^	4.550^***^	3.352^***^	1.772	-0.781	-0.915	-1.321	-3.424^**^
*COL1A1* mutation	0.226	1.557^**^	3.915^***^	2.714^**^	0.978	-0.389	-0.431	1.316	-3.249^**^
*COL1A2* mutation	0.612	1.027	1.967	1.218	-0.440	-0.366	-0.396	-2.675	0.834
Other genes mutations	0.327	1.761	1.334	2.391	2.783	-0.415	-0.497	-1.488	-1.845

OI, osteogenesis imperfecta; BSA, body surface area; PAO, proximal ascending aorta; MPA, main pulmonary artery; LAD, left atrial anteroposterior dimension; LVEDd, left ventricular end-diastolic dimension; RVEDd, right ventricular end-diastolic dimension; IVST, interventricular septal wall thickness; LVPWT, left ventricular posterior wall thickness; LVM, left ventricular mass; LVEF, left ventricular ejection fraction.

β, Regression coefficient. Adjusted for age, gender, and BSA.

^*^P < 0.05; ^**^P < 0.01; ^***^P < 0.001.

## Discussion

In this relatively large sample study, we found OI children had cardiovascular alterations, including dilated main pulmonary artery, larger internal dimensions of left hearts, and lower LVEF. These changes in cardiac structure and function seemed to be more common in patients carrying *COL1A1* mutation.

As we know, heart and blood vessels are rich in collagen, of which approximately 85% is the type I collagen, which is also the important component of valves, tendinous cords, fibrous rings, and the interventricular septum of hearts (9). Collagen fibers contribute both to maintaining the architecture and rigidity of the heart and artery (29). The reduction and disorganization of type I collagen might not only change the structure, but also affect the function of the heart (10). OI is a complex disease in which the quantity or the structure of collagen could be changed, therefore, hearts and main pulmonary arteries might be involved in OI patients (5). A previous study revealed that the heart and main pulmonary artery had decreased numbers and disorganization of collagens in perinatal lethal OI type II patients (30). In a small sample study, OI children were found to have greater dimensions of the main aorta and LVEDd than control group, but without significant change in heart function (31). Compare with control group, OI children exhibited larger LV internal dimensions and dilated main pulmonary arteries (32, 33). Our study confirmed the alterations of heart and aorta in OI children, including larger internal diameters of MPA and left heart chambers, with lower LVEF than BSA-matched healthy children. Moreover, the systolic function of the LV could be considered as its ability to generate pressure and to evacuate left ventricular blood volume. Although the smaller LVEF had no predominant clinically significance at the current stage of children and adolescents, we speculated that the cardiac function of OI patients may be changed or even be damaged as time goes on, and the patient’s cardiac function was worthy of long-term observation and follow-up.

Previous studies analyzed the correlation of cardiovascular changes with the severity of OI, and results were inconsistent. A study in 58 OI children found no cardiac abnormalities in type I OI patients, but cardiac alterations existed in type III OI children, including increased aortic root diameter and LVPW thickness (34). A recent comparative study showed that mild and moderate-severe OI patients both had larger internal diameters of the main pulmonary artery and left heart than control group, but only the moderate-severe group had lower LVEF than control group (33). In our study, echocardiographic abnormalities existed both in mild and moderate-severe OI patients, and there were no significant differences in echocardiographic measurements between these two subgroups. We speculated the divergence of the relationship between the cardiac abnormalities and OI Sillence classification may result not only from the complexity of etiology and phenotypes, but also from the insufficient sample size of OI cohort.

Few studies have evaluated the relationship of cardiac abnormalities and pathogenic gene mutations in OI patients. In this study, we found for the first time that *COL1A1* mutation of OI was correlated with larger diameters of the main pulmonary artery, larger left heart chambers and lower LVEF. We found OI children with haplo-insufficiency and triple-helical structure change of type I collagen both tended to have dilated MPA and LAD, and only patients with haplo-insufficiency of type I collagen had increased internal diameters of LVEDd than normal controls. In *Aga2* mouse model with reduced COL1A1 production, greater LVEDd and lower LVEF than wild-type mouse was found (35). Moreover, in *Oim* mouse model with defects of α2 chains of type I collagen, a disturbance in the ascending aorta was found, but without significant changes in LV structures and functions (36, 37). As type I collagen is a heterotrimer of two α1 and one α2 chains (5), we speculated that the difference of echocardiographic abnormalities might be correlated to the different changes in the structure and metabolism of type I collagen induced by various pathogenic gene mutations.

Moreover, studies reported that the cardiac valve disease was common in adult OI patients (8, 20, 38), which had not been well investigated in OI children. In a cohort of 99 OI adults and 45 healthy controls, moderate mitral regurgitation was found in 7.1% of OI patients, none in control group (39). In this study, we found OI children had significant higher percentage of mild mitral or triple valve regurgitation than control group, which was worthy of a long-term follow-up. We did not find moderate to severe valvular prolapse or regurgitation in this OI cohort, which was consistent with other studies in OI children (33). This difference of detection rate of valvular abnormalities between OI children and adults indicated that cardiovascular diseases might develop with aging in OI patients, so it was necessary to evaluate the cardiovascular structure and function of OI patients during the long-term follow-up.

We found some unspecific ECG changes in OI children, such as T-wave changes, deep Q waves, RBBB and so on. Another study also found a higher risk of unspecific ECG changes in a cohort of OI adults than normal controls (17). In this study, we found 6 OI patients with echocardiographic abnormalities, who had no obvious ECG abnormalities. We did not find the relationship between abnormalities of the echocardiography and ECG in this OI cohort. However, since the sample size of this cohort was relatively small and ECG recordings of healthy controls were not collected, it was difficult to determine whether the abnormal echocardiogram of OI patients was related to the abnormal ECG. The Framingham study indicated that asymptomatic subjects with nonspecific electrocardiographic abnormalities of ST and T-wave would have an increased risk to develop cardiovascular diseases (40). Although it is unclear the relationship of unspecific ECG changes with cardiovascular diseases, the cardiovascular changes of OI patients were still worthy of a long-term follow-up.

In this study, the cardiac structure and function of OI children were evaluated in detail, and their relationship with the clinical severity and pathogenic gene mutations were comprehensively analyzed for the first time in Asian OI patients, which expanded the understanding of the extraosseous manifestations of OI patients. However, there were some limitations in this study. We could not rule out the influence of the abnormal physical proportions of OI patients on the echocardiographic diameters and ECG recordings. Baseline characteristics and ECGs of the control group were not collected, so it was difficult to elucidate the clinical significance of the unspecific ECG changes in OI children. Since the number of different OI genotypes were disproportionate, the strength of this sub-analysis may be limited. Additionally, as the sample size was relatively small, it was difficult to investigate the effects of different gene mutations on cardiac structure and function of OI patients, especially for patients with the rare autosomal recessive gene mutations. Further researches are required to elucidate the influence of various mutation genes of OI on the cardiovascular system.

## Conclusion

Cardiovascular abnormalities are not uncommon in children with OI, which include larger dimensions of the left atrial and ventricle, dilated main pulmonary artery, and reduced left ventricular ejection fraction than healthy control. The cardiovascular system involvement seems to be more frequent in OI children with *COL1A1* mutation, and cardiovascular changes are correlated with the quantity and structural changes of type I collagen. Therefore, dynamic evaluation of cardiovascular abnormalities in OI patients has important clinical significance.

## Data availability statement

The datasets for this article are not publicly available due to concerns regarding participant/patient anonymity. Requests to access the datasets should be directed to the corresponding authors. The datasets presented in this article are not readily available because No access to raw dataset of NGS is allowed other than the Beijing Genomics institution in charge of NGS. Requests to access the datasets should be directed to https://www.genomics.cn/contact.html.

## Ethics statement

The studies involving human participants were reviewed and approved by ethic committee of Peking Union Medical College Hospital. Written informed consent to participate in this study was provided by the participants’ legal guardian/next of kin.Written informed consent was obtained from the individual(s), and minor(s)’ legal guardian/next of kin, for the publication of any potentially identifiable images or data included in this article.

## Author contributions

DZ carried out the data collection, molecular genetic studies, the sequence alignment, performed statistical analysis and drafted the manuscript. JH contributed to data collection and genetic detection. JL, and YL contributed to review and edit the manuscript. QZ, OW, YJ, XX, and WX contributed to review the manuscript. ML designed the research, interpreted the data, and revised the manuscript. All authors contributed to the article and approved the submitted version.

## Funding

This study is supported by National Key R&D Program of China (2018YFA0800801, 2021YFC2501704), CAMS Innovation Fund for Medical Sciences(CIFMS)(2021-I2M-C&T-B-007, 2021-I2M-1-051), National Natural Science Foundation of China (No.81873668, 82070908), and Beijing Natural Science Foundation (7202153).

## Acknowledgments

We appreciate the patients and their families for their participation into this study. And sincerely thank all healthy children and their parents for offering the echocardiographic profiles voluntarily.

## Conflict of interest

The authors declare that the research was conducted in the absence of any commercial or financial relationships that could be construed as a potential conflict of interest.

## Publisher’s note

All claims expressed in this article are solely those of the authors and do not necessarily represent those of their affiliated organizations, or those of the publisher, the editors and the reviewers. Any product that may be evaluated in this article, or claim that may be made by its manufacturer, is not guaranteed or endorsed by the publisher.
